# Lutein Modulates Stress-Responsive Signaling Pathways in THLE-2 Human Hepatocytes Under Intestinal Failure–Associated Liver Disease Conditions

**DOI:** 10.3390/molecules31091413

**Published:** 2026-04-24

**Authors:** Izabela Żółnowska, Violetta Krajka-Kuźniak, Marta Belka, Grzegorz Adamek, Maciej Stawny

**Affiliations:** 1 Poznan University of Medical Sciences, Department of Pharmaceutical Chemistry, Rokietnicka 3, 60-806 Poznan, Poland; 2 Poznan University of Medical Sciences, Doctoral School, Bukowska 70, 60-812 Poznan, Poland; mbelka@ump.edu.pl; 3 Poznan University of Medical Sciences, Department of Pharmaceutical Biochemistry, Rokietnicka 3, 60-806 Poznan, Poland; vkrajka@ump.edu.pl; 4 Faculty of Materials Engineering and Technical Physics, Institute of Materials Science and Engineering, Poznan University of Technology, Jana Pawła II No. 24, 61-139 Poznan, Poland; grzegorz.adamek@put.poznan.pl

**Keywords:** non-provitamin A carotenoids, xanthophylls, parenteral nutrition, parenteral nutrition-associated liver disease, clinical nutrition, nanoparticles, nanocarriers, drug delivery, human serum albumin

## Abstract

Intestinal dysfunction and parenteral nutrition (PN) can trigger a spectrum of liver disorders collectively referred to as intestinal failure-associated liver disease (IFALD), for which therapeutic options remain limited. In the present study, we investigated the modulatory effects of the bioactive xanthophyll carotenoid lutein in an in vitro IFALD model utilizing human THLE-2 hepatocytes exposed to lipopolysaccharide and Intralipid to mimic PN–associated inflammatory and metabolic stress. Because lutein is poorly water-soluble and patients receiving PN lack enteral intake of this compound, we also evaluated the cyto- and hemocompatibility of a human serum albumin–based lutein nanoformulation developed to enable intravenous administration. A bead-based multiplex immunoassay revealed that lutein attenuated dysregulation of inflammatory and metabolic signaling by modulating total and phosphorylated levels of MAPKs, NF-κB, Akt, STAT5, CREB, and p70S6K. Lutein also affected lipid metabolism–related gene expression, decreasing *SREBF2* and restoring *ABCA1* and *PRKAA2* mRNA toward control levels, as determined by qPCR. Nanoformulated lutein, with a mean particle size of approximately 160 nm, was non-toxic in THLE-2 cells and exhibited hemocompatibility in a human erythrocyte hemolysis assay. Together, our findings provide both biological and technological rationale for further exploration of lutein-based strategies to mitigate IFALD in patients receiving PN.

## 1. Introduction

Intestinal failure–associated liver disease (IFALD) refers to a spectrum of liver diseases, ranging from cholestasis and steatohepatitis to cirrhosis and liver failure, that develop as a consequence of intestinal failure in the absence of other primary causes of liver injury. Its pathogenesis is complex and often involves impaired gut barrier integrity, leading to bacterial endotoxin translocation into the circulation, hepatic inflammation, and ultimately the progression of IFALD [1,2]. Moreover, the disease course may be influenced by the composition of parenteral nutrition (PN), a vital intervention for patients who cannot be fed via the gastrointestinal tract. Although it provides essential macro- and micronutrients, PN may adversely affect liver function due to an unfavorable omega-3-to-omega-6 fatty acid ratio and limited antioxidant supply. In addition, high levels of pro-cholestatic phytosterols in PN lipid emulsions have also been associated with the development of liver injury [2,3].

IFALD pathogenesis involves the gut–liver axis and several hepatic cell populations, including Kupffer cells, hepatic stellate cells, and hepatocytes [4,5]. Studies conducted across diverse experimental settings have aimed to clarify how different triggers and dysregulated signaling pathways contribute to disease progression. While IFALD models vary in the injurious stimuli applied and the cell types used [6,7,8], different approaches provide insight into selected aspects of the disease. However, their common limitation is the use of non-human experimental systems or transformed hepatoma cell lines, which may not fully reflect human liver metabolism or nutrient responsiveness [9,10]. Therefore, further research is warranted to elucidate the mechanisms underlying the multifactorial pathogenesis of IFALD. In this context, non-tumorigenic human hepatocytes offer a relevant experimental model for investigating hepatocellular signaling responses to IFALD-related inflammatory and metabolic stressors.

Despite ongoing efforts to mitigate IFALD, including modifications to the composition of PN admixtures [11], the condition remains a clinically relevant problem in patients with intestinal failure. The prevalence of IFALD varies widely across populations, with estimates of 40–60% in pediatric patients, up to 85% in neonates, and 15–40% in adults reported in the literature, reflecting differences in diagnostic criteria and patient characteristics [12]. Therefore, additional interventions are needed for patients dependent on PN to prevent and treat IFALD.

We hypothesized that natural plant bioactives, including carotenoids such as lutein, with well-documented health-promoting properties [13], could offer a promising strategy for mitigating IFALD. Lutein is a xanthophyll abundant in green leafy vegetables that cannot be synthesized by the human body, making the oral diet its only source [13]; therefore, patients with intestinal failure, who are unable to receive enteral nutrition, are deprived of dietary lutein. Owing to its potent antioxidant and anti-inflammatory properties, lutein has been extensively investigated for its protective effects in ocular [14], cardiometabolic [15], and neurodegenerative [16] disorders. Increasing evidence also indicates its hepatoprotective potential, with studies demonstrating its ability to attenuate oxidative stress, modulate inflammatory signaling pathways, and regulate lipid metabolism in experimental models of liver injury [17,18,19]. However, despite its favorable biological profile, the role of lutein and other carotenoids in IFALD has, to our knowledge, not yet been explored. Moreover, lutein’s poor water solubility poses a practical challenge for its effective application, underscoring the need for appropriate delivery systems [20].

In the present study, we employed our previously developed in vitro IFALD model [21] utilizing THLE-2 human hepatocytes to investigate the modulatory effects of lutein under conditions mimicking PN-associated liver injury. In this setting, lutein effectively attenuated IFALD-related inflammatory activation and disturbances in metabolic signaling pathways. To allow potential intravenous administration of lutein in patients dependent on PN, we developed a PN-compatible human serum albumin–based lutein nanoformulation [22]. Albumin, the most abundant plasma protein and a natural carrier of endogenous and exogenous compounds, offers excellent biocompatibility as a drug delivery platform [23] and has enabled the development of a formulation with favorable physicochemical properties for parenteral administration. In this study, we evaluated its cytotoxicity and hemolytic activity using THLE-2 cells and human erythrocytes, respectively. The formulation demonstrated favorable safety characteristics in both hepatocyte metabolic activity and erythrocyte compatibility assays. Together, our findings provide both a biological rationale and technological feasibility supporting lutein as a potential adjunct strategy to mitigate IFALD in patients receiving PN.

## 2. Results and Discussion

### 2.1. IFALD-Related Triggers Induce Inflammatory and Metabolic Signaling Alterations in THLE-2 Hepatocytes

**Figure 1:**
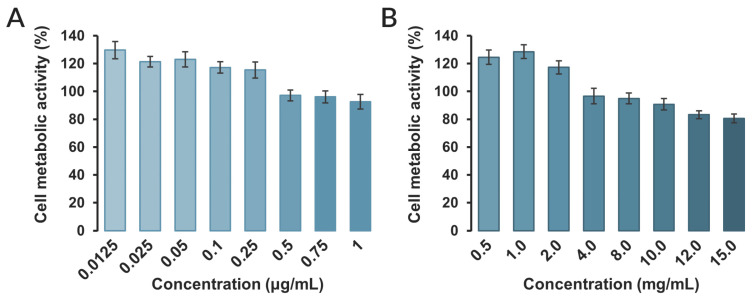
Effect of lipopolysaccharide (LPS) and Intralipid on the metabolic activity of THLE-2 cells after 24 h of incubation. Cells were treated with a range of concentrations of LPS (**A**) or Intralipid (**B**), and metabolic activity was assessed using the 3-(4,5-dimethylthiazol-2-yl)-2,5-diphenyltetrazolium bromide (MTT) assay. Results are expressed as a percentage relative to untreated control cells (set as 100%). Data are presented as mean ± SEM (*n* = 3).

To capture the multifactorial nature of IFALD, we employed an in vitro model using human THLE-2 hepatocytes exposed to two key pathogenic stimuli associated with PN–related liver injury. Cells were treated with lipopolysaccharide (LPS), which represents circulating bacterial endotoxins, and Intralipid, a soybean oil–based lipid emulsion used in PN, characterized by a high omega-6-to-omega-3 fatty acid ratio. The concentrations of LPS (0.1 µg/mL) and Intralipid (10 mg/mL) were selected based on previously reported studies [7,8,24,25]. The 3-(4,5-dimethylthiazol-2-yl)-2,5-diphenyltetrazolium bromide (MTT) assay was performed to confirm that the chosen conditions did not adversely affect the metabolic activity of THLE-2 cells, as a decrease in metabolic activity may reflect cytotoxic effects. The results (Figure 1) showed no marked decrease in metabolic activity under the selected conditions, confirming their suitability for further experiments.

Changes in inflammation- and metabolism-related signaling were assessed by analyzing total and phosphorylated (activated) levels of selected cellular components following exposure to each stimulus individually or in combination (hereafter referred to as the IFALD model). Phosphorylation of signaling proteins is often evaluated at early time points (e.g., 30–60 min) as a rapid cellular response to stimulation. However, an in vitro study by Miskolci et al. [26] demonstrated that a key regulator of inflammatory responses, nuclear factor κB (NF-κB), may remain persistently activated under continuous stimulation, leading to sustained production of proinflammatory mediators and the progression of various diseases. Additionally, exposure to endotoxins for 18 h has been shown to induce time- and dose-dependent increases in the phosphorylation of mitogen-activated protein kinases (MAPKs), including p38 and extracellular signal–regulated kinases 1/2 (ERK1/2), indicating pathway activation at later time points [27]. Another example of such an approach is the study by Rao et al. [28], in which the phosphorylation of p38 and ERK1/2 was assessed in primary human monocytes at early time points (15 min and 1 h) and late time points (24 h) following LPS stimulation. Moreover, in a study utilizing combined fatty acid and high-glucose treatment, incubation for ≥24 h has been used to establish in vitro insulin resistance models, characterized by decreased protein kinase B (Akt) phosphorylation [29], supporting the relevance of extended incubation periods for assessing sustained metabolic signaling disturbances. Therefore, in the present study, THLE-2 cells were exposed to IFALD-related stimuli for 24 h to capture inflammatory and metabolic responses that more closely correspond to sustained hepatic exposure to bacterial endotoxins and lipotoxic conditions associated with IFALD, rather than the initial activation peaks. Importantly, this experimental design also allows evaluation of the modulatory effects of lutein under prolonged proinflammatory and lipotoxic stimulation, better reflecting its potential therapeutic relevance. Notably, during the applied 24 h incubation, changes in total protein levels may occur alongside alterations in phosphorylation status, which should be taken into account when interpreting phosphorylation-related data. Therefore, phosphorylated and total protein levels were analyzed separately to assess both changes in overall protein expression and differences in the abundance of phosphorylated forms relative to control conditions. This approach is consistent with previous biological studies that assessed phosphorylated and total protein levels separately to distinguish changes in protein activation from alterations in protein expression [30,31].

**Figure 2:**
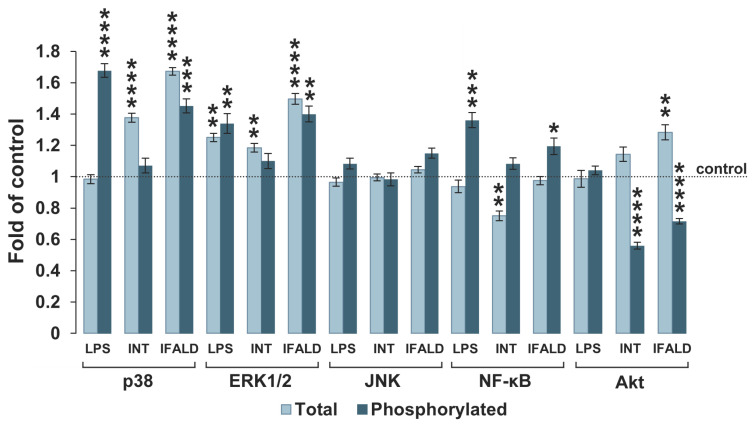
Effects of LPS (0.1 µg/mL), Intralipid (INT, 10 mg/mL), or their combination (IFALD model) on signaling pathways in THLE-2 cells after 24 h of exposure. Total and phosphorylated levels of p38, extracellular signal–regulated kinases 1/2 (ERK1/2), c-Jun N-terminal kinase (JNK), nuclear factor κB (NF-κB), and protein kinase B (Akt) were quantified based on fluorescence intensity using the MAGPIX system. Data are expressed as fold change relative to untreated controls (mean ± SEM, *n* = 3). Statistical significance was determined using Dunnett’s test. * *p* < 0.05, ** *p* < 0.01, *** *p* < 0.001, and **** *p* < 0.0001 vs. control. The horizontal dotted line indicates the control levels.

As shown in Figure 2, combined exposure to LPS and Intralipid elicited a distinct hepatocellular signaling response compared with either stimulus alone. Among MAPKs, p38 and ERK1/2 displayed the most pronounced changes in total and phosphorylated protein levels in the IFALD model. LPS exposure significantly increased p38 phosphorylation without markedly affecting its total level, indicating pathway activation. In contrast, Intralipid increased total p38 levels without a concomitant rise in phosphorylation. Combined exposure resulted in increased total and phosphorylated p38 levels, suggesting concurrent increases in kinase abundance and activation under IFALD-like conditions.

A similar pattern was observed for ERK1/2. LPS increased both total and phosphorylated ERK1/2 levels, whereas Intralipid primarily elevated total ERK1/2 without substantial activation. The combined treatment further enhanced ERK1/2 abundance and phosphorylation. In contrast, neither LPS nor Intralipid, alone or in combination, significantly affected the total or phosphorylated levels of c-Jun N-terminal kinase (JNK), indicating selective engagement of MAPK pathways in this model.

The observed modulation of p38 and ERK1/2 signaling is consistent with previous reports indicating that endotoxin exposure and omega-6 fatty acid–derived mediators can influence MAPK regulation. LPS is a well-established activator of Toll-like receptor 4 (TLR4)–dependent signaling pathways [32], whereas omega-6 fatty acids present in soybean oil–based lipid emulsions may be enzymatically converted into arachidonic acid, which has been implicated in the modulation of kinase activity [33,34]. While phytosterols also present in soybean oil have been associated with IFALD-related inflammation, available evidence indicates that they do not elicit a proinflammatory response in hepatocytes, but rather act through other liver cell types [35]. Accordingly, the inflammation-related signaling changes observed in the present THLE-2 hepatocyte-based model are unlikely to be directly attributable to phytosterols.

Our findings are in line with previous reports demonstrating that the fatty acid composition of co-administered parenteral lipid emulsions strongly influences the inflammatory response of hepatocytes to LPS. In a hepatocyte-based model employed by Ventro et al. [8], supplementation with an omega-3–rich lipid emulsion (Omegaven) attenuated LPS-induced ERK1/2 activation, whereas the present study observed enhanced ERK1/2 abundance and phosphorylation following combined exposure to LPS and the omega-6–rich Intralipid. These results support the concept that the balance of omega-6 and omega-3 fatty acids critically modulates MAPK-dependent inflammatory signaling in hepatocytes under PN-related stress conditions. Notably, despite activation of p38 and ERK1/2, JNK signaling remained largely unaffected in our model, consistent with reports of antagonistic interactions within the MAPK family, in which p38 activation can suppress JNK signaling in hepatocytes [36].

Analysis of NF-κB signaling further highlighted the complexity of pathway crosstalk in the studied model. LPS significantly increased NF-κB phosphorylation, as expected for canonical TLR4-mediated activation [37]. In contrast, Intralipid reduced total NF-κB levels while maintaining phosphorylation near control levels, and the combined IFALD model showed an increase in NF-κB phosphorylation, although the effect was less pronounced than in the LPS group. These findings suggest that lipid-mediated stress may interfere with sustained NF-κB signaling despite the presence of an inflammatory stimulus. The attenuated NF-κB response may be linked to disturbances in the phosphoinositide 3-kinase/Akt pathway. Intralipid markedly reduced Akt phosphorylation, an effect that persisted in the combined IFALD model despite increased total Akt levels. Since Akt enhances the activity of NF-κB [38], impaired Akt signaling may limit downstream NF-κB activation. Talukdar et al. [34] showed that arachidonic acid can inhibit Akt phosphorylation via p38-dependent serine phosphorylation of insulin receptor substrate-1, leading to insulin resistance–like signaling alterations in hepatocytes. The activation of p38 observed here may therefore contribute to impaired Akt signaling and downstream attenuation of NF-κB activation.

**Figure 3:**
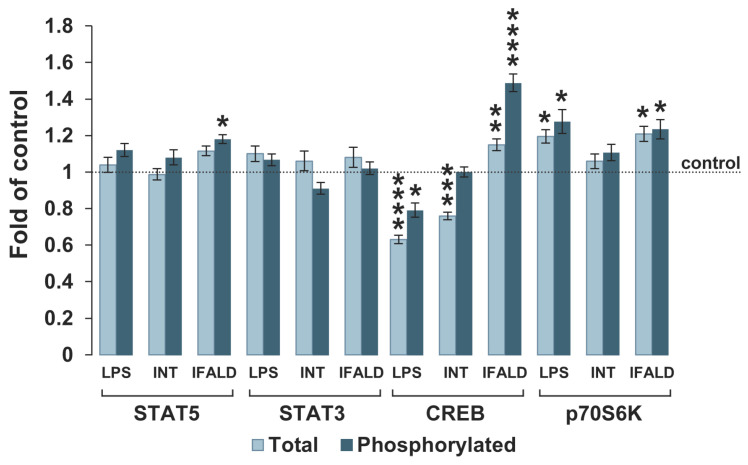
Effects of LPS (0.1 µg/mL), Intralipid (INT, 10 mg/mL), or their combination (IFALD model) on signaling pathways in THLE-2 cells after 24 h of exposure. Total and phosphorylated levels of signal transducer and activator of transcription 3 and 5 (STAT3 and STAT5), cAMP response element-binding protein (CREB), and 70 kDa ribosomal protein S6 kinase (p70S6K) were quantified based on fluorescence intensity using the MAGPIX system. Data are expressed as fold change relative to untreated controls (mean ± SEM, *n* = 3). Statistical significance was determined using Dunnett’s test. * *p* < 0.05, ** *p* < 0.01, *** *p* < 0.001, and **** *p* < 0.0001 vs. control. The horizontal dotted line indicates the control levels.

Beyond MAPKs, NF-κB, and Akt, several other signaling regulators were assessed. Signal transducers and activators of transcription 3 and 5 (STAT3 and STAT5) are involved in cytokine-mediated signaling and the regulation of hepatic metabolism and survival [39,40], cAMP response element-binding protein (CREB) controls lipid and glucose metabolism [41,42], whereas 70 kDa ribosomal protein S6 kinase (p70S6K) is involved in glucose homeostasis preservation, cell growth, and survival [43]. The total and phosphorylated levels of these factors in THLE-2 cells treated with LPS, Intralipid, or their combination are shown in Figure 3.

Phosphorylation of STAT5 was significantly increased only upon combined exposure to LPS and Intralipid, whereas each stimulus alone had no marked effect, suggesting that STAT5 activation may require both inflammatory and lipid-derived triggers. In contrast, STAT3 levels remained largely unchanged across all experimental conditions. Ghosh et al. [44] reported the involvement of STAT3 in farnesoid X receptor (FXR)–mediated anticholestatic signaling in IFALD; however, the lack of pronounced STAT3 modulation suggests that this regulatory axis is not strongly dysregulated in the present model.

CREB signaling was differentially regulated depending on the applied stimulus. Exposure to Intralipid or LPS alone significantly reduced total CREB levels, with a concomitant decrease in CREB phosphorylation observed following LPS treatment. In contrast, combined exposure elevated total CREB level, accompanied by increased CREB phosphorylation. CREB integrates metabolic and inflammatory signaling in hepatocytes in a context-dependent manner [45,46]; thus, the enhanced CREB activation observed under concurrent lipid- and inflammation-related stress may reflect a specific adaptive response of THLE-2 hepatocytes to IFALD-like conditions.

p70S6K signaling was up-regulated in response to LPS exposure, leading to significant increases in total and phosphorylated p70S6K levels, whereas Intralipid alone had no marked effect. Combined treatment with LPS and Intralipid also resulted in increased p70S6K phosphorylation and total levels. Notably, combined exposure did not further enhance this effect beyond that observed with LPS alone, indicating that p70S6K up-regulation in this model is primarily driven by the endotoxin.

Taken together, these results demonstrate that exposure to LPS and a soybean oil–based lipid emulsion elicits a distinct hepatocellular signaling response compared with either stimulus alone, characterized by coordinated modulation of inflammatory and metabolic pathways in human THLE-2 hepatocytes. These findings provide mechanistic insight into intracellular signaling alterations in non-tumorigenic human hepatocytes, complementing existing IFALD studies that have largely relied on transformed hepatoma cell lines or non-human experimental models, which exhibit distinct metabolic and signaling profiles.

At the same time, the present approach focuses on hepatocyte-autonomous responses and does not account for intercellular interactions or gut–liver axis feedback mechanisms that contribute to IFALD pathogenesis in vivo. Future studies employing more complex experimental systems, such as three-dimensional hepatic models, may further extend these observations by capturing additional aspects of cellular crosstalk. Nevertheless, the THLE-2 hepatocyte-based IFALD model established here provides a controlled framework for examining the effects of defined IFALD-related stimuli and was subsequently used to assess lutein-mediated modulation of IFALD-associated cellular responses.

### 2.2. Lutein Modulates Dysregulated Signaling Pathways in the In Vitro IFALD Model

**Figure 4:**
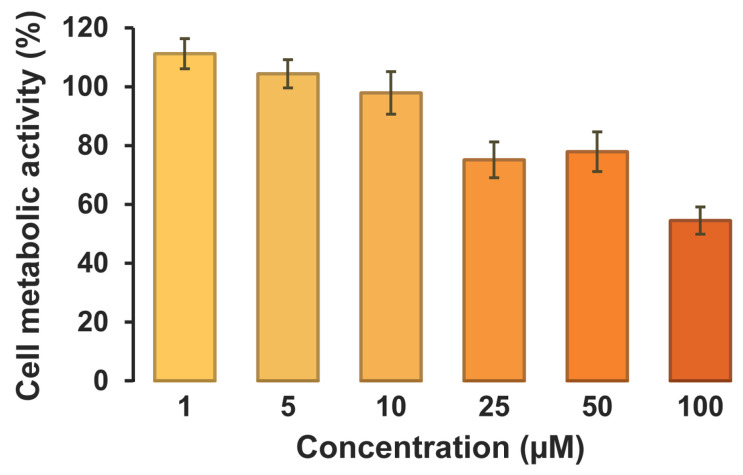
Effect of lutein on THLE-2 cell metabolic activity after 24 h of exposure. Cell metabolic activity was assessed using the MTT assay across a range of lutein concentrations (1–100 µM) and expressed as a percentage relative to untreated control cells. Data are presented as mean ± SEM (*n* = 3).

To define lutein concentrations suitable for mechanistic studies, THLE-2 cell metabolic activity after lutein exposure was evaluated using the MTT assay. The results revealed a concentration-dependent reduction in metabolic activity (Figure 4). Although metabolic activity decreased with increasing lutein concentration, the half-maximal inhibitory concentration (IC50) was not reached within the tested range. Based on these results, lutein concentrations of 10 and 25 µM were selected for subsequent experiments to assess signaling modulation in THLE-2 cells under IFALD-like conditions.

**Figure 5:**
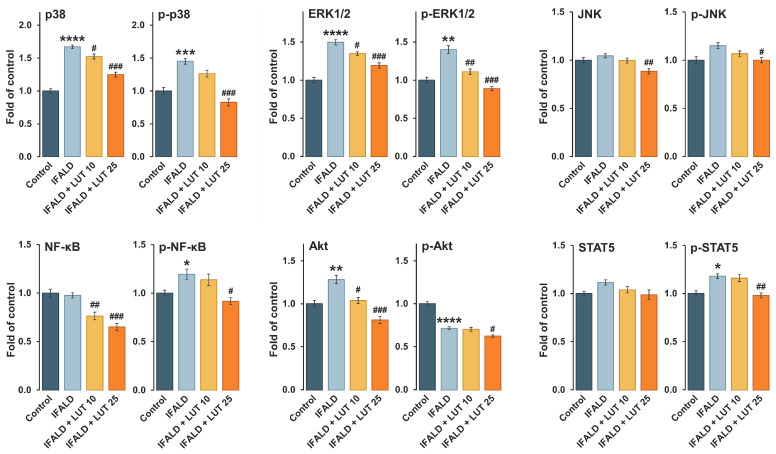
Effects of lutein on cellular signaling components in the in vitro IFALD model. THLE-2 cells were treated with LPS (0.1 µg/mL) and Intralipid (10 mg/mL) to mimic IFALD-like conditions and co-treated with lutein (LUT) at concentrations of 10 or 25 µM for 24 h. Total and phosphorylated levels of p38, ERK1/2, JNK, NF-κB, Akt, and STAT5 were quantified based on mean fluorescence intensity using the MAGPIX System. Data are expressed as fold change relative to untreated control cells (mean ± SEM, *n* = 3). Statistical significance was determined using Dunnett’s test. * *p* < 0.05, ** *p* < 0.01, *** *p* < 0.001, and **** *p* < 0.0001 vs. control; # *p* < 0.05, ## *p* < 0.01, and ### *p* < 0.001 vs. IFALD.

**Figure 6:**
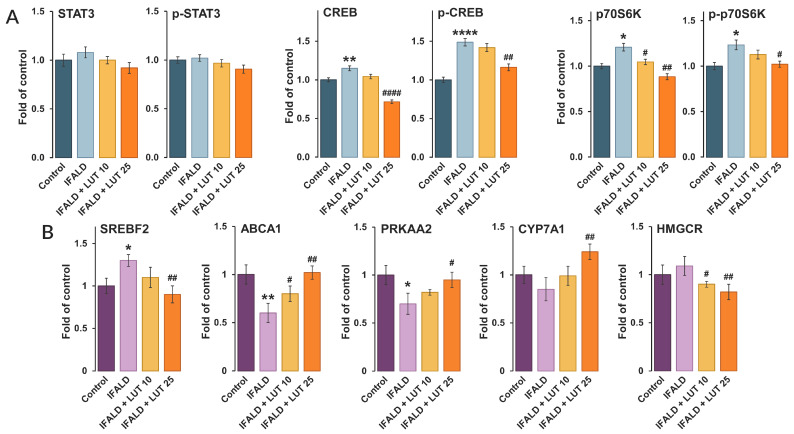
Effects of lutein on cellular signaling components in the in vitro IFALD model. THLE-2 cells were treated with LPS (0.1 µg/mL) and Intralipid (10 mg/mL) to mimic IFALD-like conditions and co-treated with lutein (LUT) at concentrations of 10 or 25 µM for 24 h. (**A**) Total and phosphorylated levels of STAT3, CREB, and p70S6K, quantified based on mean fluorescence intensity using the MAGPIX System. (**B**) mRNA expression levels of sterol regulatory element-binding protein 2 (*SREBF2*), ATP-binding cassette subfamily A member 1 (*ABCA1*), AMP-activated protein kinase α2 (*PRKAA2*), cholesterol 7 α-hydroxylase (*CYP7A1*), and 3-hydroxy-3-methylglutaryl-CoA reductase (*HMGCR*), determined by quantitative real-time polymerase chain reaction (qPCR). Data are expressed as fold change relative to untreated control cells (mean ± SEM, *n* = 3). Statistical significance was determined using Dunnett’s test. * *p* < 0.05, ** *p* < 0.01, **** *p* < 0.0001 vs. control; # *p* < 0.05, ## *p* < 0.01, and #### *p* < 0.0001 vs. IFALD.

Lutein treatment modulated the total levels and activity of multiple cellular components altered under IFALD-like conditions (Figure 5 and Figure 6A). In particular, lutein reduced both total and phosphorylated levels of stress-activated MAPKs p38 and ERK1/2, with more pronounced effects observed at the higher concentration. Despite the absence of significant JNK changes in the IFALD model, lutein decreased JNK levels at a concentration of 25 µM, indicating a broader modulatory effect on MAPK-associated signaling. However, the decrease in total protein levels observed at 25 µM may be partially influenced by the reduction in cell metabolic activity (~25%), which may reflect a decrease in cell number, and should be taken into account when interpreting these results.

Lutein exerted concentration-dependent effects on inflammatory signaling, reducing both total and phosphorylated levels of NF-κB and Akt. In parallel, lutein reduced STAT5 phosphorylation, which was increased in the IFALD model, while STAT3 signaling remained largely unaffected. Additionally, lutein modulated CREB and p70S6K signaling, reducing IFALD-associated increases in their total and phosphorylated levels.

To further evaluate whether lutein influences metabolic homeostasis at the transcriptional level, the expression of selected genes involved in lipid and cholesterol metabolism was assessed by quantitative real-time polymerase chain reaction (qPCR) (Figure 6B). These included sterol regulatory element-binding protein 2 (SREBP2; *SREBF2*), ATP-binding cassette subfamily A member 1 (*ABCA1*), AMP-activated protein kinase α2 (AMPKα2; *PRKAA2*), cholesterol 7 α-hydroxylase (*CYP7A1*), and 3-hydroxy-3-methylglutaryl-CoA reductase (*HMGCR*).

In the IFALD model, *SREBF2* was up-regulated compared with control cells. Notably, co-treatment with lutein at 25 µM significantly reduced *SREBF2* mRNA levels relative to the IFALD group. In contrast, IFALD-like conditions significantly suppressed *ABCA1* and *PRKAA2*, whereas lutein restored their mRNA expression toward control values, supporting improved regulation of cholesterol efflux and lipid metabolism.

No statistically significant differences were observed in the expression of *CYP7A1* and *HMGCR* between IFALD and control cells, indicating that these metabolic regulators were not markedly dysregulated in the present THLE-2 hepatocyte-based model. This may reflect the limitations of the simplified in vitro system, as bile acid synthesis regulated by CYP7A1 is strongly influenced by enterohepatic feedback mechanisms and gut-derived signaling, which are absent in isolated hepatocyte cultures. However, lutein supplementation significantly increased *CYP7A1* and decreased *HMGCR* expression compared with the IFALD group, suggesting lutein-mediated modulation of bile acid-related transcriptional responses and cholesterol biosynthesis under IFALD-like conditions.

**Scheme 1:**
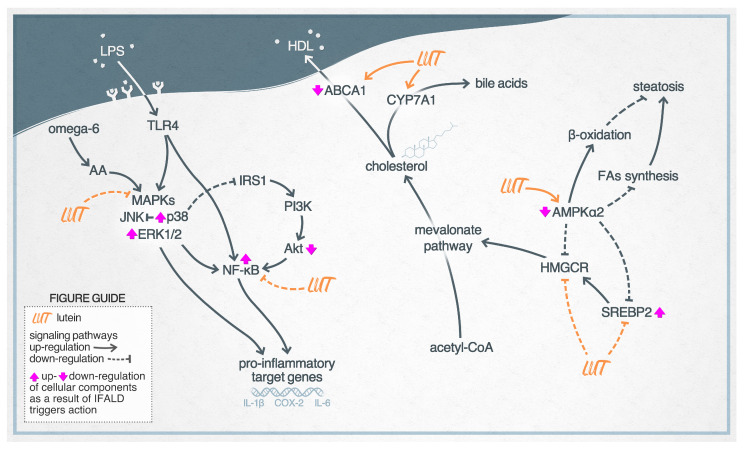
Proposed mechanism of action of lutein in THLE-2 hepatocytes exposed to IFALD-related triggers—LPS and omega-6-rich Intralipid. AA, arachidonic acid; TLR4, Toll-like receptor 4; IRS1, insulin receptor substrate 1; PI3K, phosphoinositide 3-kinase; IL-1β, interleukin-1β; COX-2, cyclooxygenase-2; IL-6, interleukin-6; HDL, high-density lipoprotein; FAs, fatty acids.

The presented results demonstrate that lutein regulates multiple cellular components altered under IFALD-like conditions in THLE-2 hepatocytes (Scheme 1). Among them, p38 MAPK may be particularly important, given its potential role in coordinating stress-responsive signaling through interactions with JNK and Akt. Therefore, alterations in p38 activity may contribute to the broader signaling changes observed in the study. In the IFALD model, combined exposure to inflammatory and lipid-derived stimuli markedly increased both total and phosphorylated p38 levels, and lutein attenuated these elevations. Interestingly, lutein reduced JNK levels, despite the absence of significant IFALD-induced changes in this regulator, suggesting that lutein may influence inflammatory signaling beyond pathways directly dysregulated by IFALD-like conditions. Moreover, lutein significantly reduced the levels of NF-κB, a key regulator of the inflammatory response. In addition, lutein modulated gene expression, total protein levels, and activation of selected lipid metabolism–related cellular components. However, reduced cell metabolic activity at 25 µM may have influenced the observed effects at this concentration. Overall, these findings support lutein’s role in maintaining hepatocellular metabolic homeostasis under IFALD-like conditions.

The protective effects of lutein have been described in various experimental models of liver injury, including metabolic and inflammatory conditions such as non-alcoholic fatty liver disease, where lutein has been shown to regulate lipid metabolism, inflammatory signaling, and oxidative stress [19,47,48,49,50,51]. Mechanistically, lutein is a well-recognized antioxidant [52], owing to the extended conjugation of double bonds within its polyene structure, which enables efficient scavenging of reactive radicals [53]. In addition, lutein has been reported to enhance antioxidant defense systems by regulating nuclear factor erythroid 2-related factor 2 (Nrf2)-dependent pathways and the expression of antioxidant enzymes [50,54,55,56]. Given that oxidative stress is considered an important contributor to IFALD pathogenesis [3], the observed effects of lutein on hepatocellular signaling may, at least in part, be linked to its antioxidant activity, although oxidative stress was not directly assessed in the present study. Specifically, we did not quantify intracellular reactive oxygen species (ROS) levels nor assess canonical antioxidant response readouts (e.g., lipid peroxidation products or expression/activity of enzymes such as superoxide dismutase and catalase). Therefore, the contribution of redox modulation to the observed attenuation of p38/NF-κB signaling should be interpreted as a working hypothesis rather than a demonstrated mechanism. Nevertheless, this interpretation is biologically plausible because both p38 MAPK and NF-κB are responsive to oxidative cues and ROS-dependent upstream events that can amplify inflammatory transcriptional programs.

Beyond redox-related mechanisms, lutein has also been shown to attenuate inflammatory signaling by reducing NF-κB and p38 MAPK activation, as well as lowering the expression of pro-inflammatory cytokines in hepatic models [19,51,57,58]. These reported mechanisms are consistent with the attenuation of inflammation-related signaling observed in the present in vitro IFALD model.

While our study’s findings support the biological relevance of lutein in the context of IFALD, its potential application depends on practical considerations related to delivery, with the route of administration being a critical factor. Patients with intestinal failure require PN and are unable to rely on enteral nutrition, necessitating intravenous administration of any adjunctive compounds. However, lutein is characterized by poor water solubility and physicochemical instability [59], which preclude its direct use in parenteral formulations. These limitations underscore the need for an appropriate delivery system that enables safe systemic administration of lutein. With this in mind, we developed a lutein nanoformulation using serum albumin as a carrier matrix [22], which prompted further investigation of its biological compatibility.

### 2.3. Physicochemical Characterization of Lutein Nanoformulation

**Figure 7:**
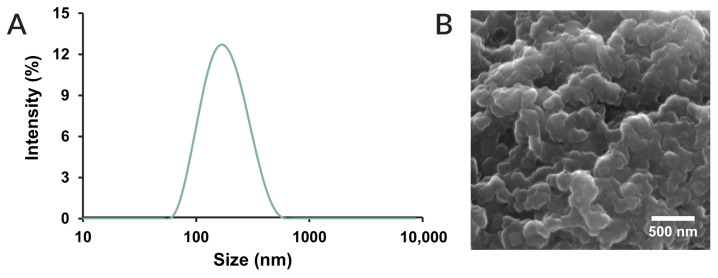
Size and morphology of Albumin-Lutein Nanosuspension (AlbLuteN). (**A**) Intensity-weighted size distribution curve determined by dynamic light scattering. (**B**) Representative scanning electron microscopy (SEM) image.

A human serum albumin–based nanoformulation loaded with lutein (Albumin-Lutein Nanosuspension, AlbLuteN) was prepared using a modified nanoparticle albumin-bound (nab™) technology [60]. Dynamic light scattering analysis showed an intensity-weighted mean hydrodynamic diameter (Z-average) of the nanoparticles measured at 160.47 ± 3.79 nm with a narrow size distribution (polydispersity index (PDI) = 0.178 ± 0.009) (Figure 7A). Scanning electron microscopy (SEM) analysis confirmed the nanoscale size and revealed a roughly spherical morphology of the AlbLuteN particles (Figure 7B). The obtained particle size below 200 nm and the low PDI indicate a uniform nanoparticle population suitable for intravenous administration. Nanoparticles in this size range are generally considered compatible with systemic circulation [61], and particle homogeneity is important for predictable biological performance [62].

AlbLuteN exhibited a negative surface charge with a zeta potential of −35.07 ± 3.49 mV. The negative charge observed is characteristic of serum albumin and contributes to electrostatic stabilization of the colloidal system. Such surface properties are advantageous for maintaining dispersion stability by limiting particle aggregation [61].

The drug loading of lutein in the albumin matrix was 4.7 ± 0.5%, indicating efficient incorporation of lutein within the protein matrix despite its hydrophobic nature and poor aqueous solubility. This loading capacity enables the delivery of biologically relevant amounts of lutein.

Human serum albumin is a biocompatible, clinically established drug carrier, naturally present in human plasma and widely recognized for its favorable safety profile and intrinsic ability to bind and transport hydrophobic ligands in the bloodstream [23]. These properties make serum albumin-based nanoparticles particularly suitable for applications requiring parenteral administration under conditions relevant to PN. Given the intended intravenous infusion, the interaction of AlbLuteN with blood components was next investigated.

### 2.4. Hemocompatibility of AlbLuteN

**Figure 8:**
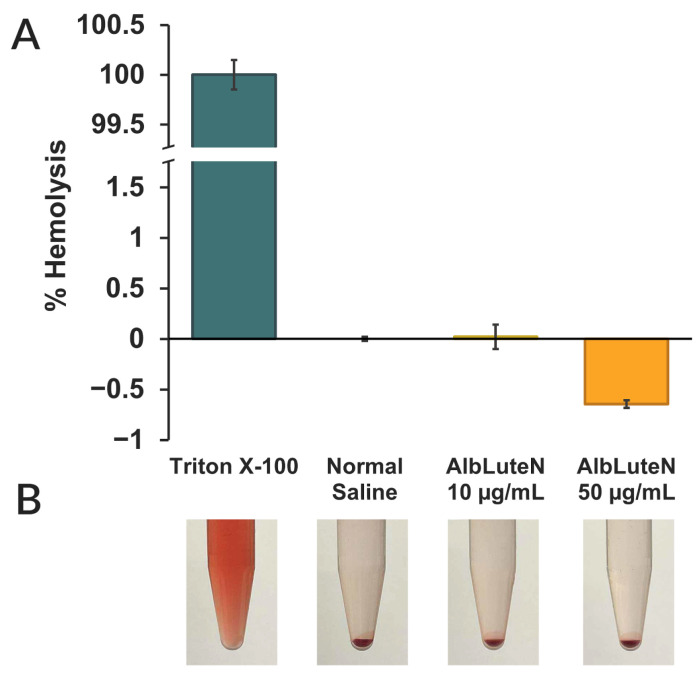
Hemocompatibility of AlbLuteN. (**A**) % hemolysis induced by 10% Triton X-100 (positive control), normal saline (negative control), and AlbLuteN at concentrations of 10 and 50 µg/mL, expressed as total nanoformulation concentration. Samples were incubated with a 1% erythrocyte suspension for 1 h, centrifuged, and the absorbance of the resulting supernatants was measured at 577 nm. Data are presented as mean ± SD (*n* = 3). (**B**) Representative photographs of sample tubes.

Hemocompatibility is a key requirement for drug delivery systems intended for systemic administration [63]. Therefore, the hemolytic activity of AlbLuteN at two concentrations (10 and 50 µg/mL, expressed as total nanoformulation concentration) was evaluated using an erythrocyte-based assay, with Triton X-100 and normal saline serving as positive and negative controls, respectively. The results of the hemolysis assay are presented in Figure 8. As expected, Triton X-100 induced hemolysis, whereas the negative control exhibited negligible hemolytic activity. Importantly, AlbLuteN did not induce detectable hemolysis at either concentration tested. In both cases, hemolysis values remained far below the widely accepted safety threshold of 5% [64], indicating excellent hemocompatibility of the formulation. Notably, at the higher concentration, the level of free hemoglobin released from damaged erythrocytes was even lower than that observed for the negative control, resulting in a slightly negative hemolysis value (−0.64 ± 0.04%). A similar observation has been previously reported for apatite nanoparticles and attributed to the protective effect of the colloid against cell denaturation [65].

To exclude the possibility that the low and negative hemolysis values resulted from assay interference rather than true hemocompatibility, a hemoglobin binding/co-sedimentation control was performed. AlbLuteN (at a final concentration corresponding to the 50 µg/mL sample) was added to the free hemoglobin obtained from the positive control, mixed, and centrifuged in the same way as the tested samples. The absorbance of the resulting supernatant was compared with that of free hemoglobin processed under identical conditions, with saline added instead of AlbLuteN. No statistically significant difference in absorbance was observed between these two conditions, indicating that AlbLuteN did not bind hemoglobin or promote its co-sedimentation during centrifugation. This confirms that the hemolysis values observed for AlbLuteN-treated erythrocytes were not falsely underestimated due to hemoglobin–nanoparticle interactions, but instead reflect the formulation’s true hemocompatibility.

Albumin is widely recognized for its favorable biocompatibility and has been extensively used in drug delivery systems [23]. The observed trend toward reduced hemolysis in the presence of AlbLuteN may therefore reflect a stabilizing effect of the albumin-based nanocarrier on erythrocytes. Such behavior is consistent with previous reports describing albumin-based formulations as highly hemocompatible, even at relatively high concentrations [66,67,68].

Overall, the hemolysis assay results demonstrate that AlbLuteN exhibits excellent hemocompatibility at both tested concentrations, supporting its suitability for biomedical applications involving direct blood contact. To further support its suitability for systemic administration, AlbLuteN interactions with cells were evaluated as the next step in the safety assessment; therefore, its cytocompatibility was investigated in THLE-2 human hepatocytes.

### 2.5. Cytocompatibility of AlbLuteN with THLE-2 Hepatocytes

**Figure 9:**
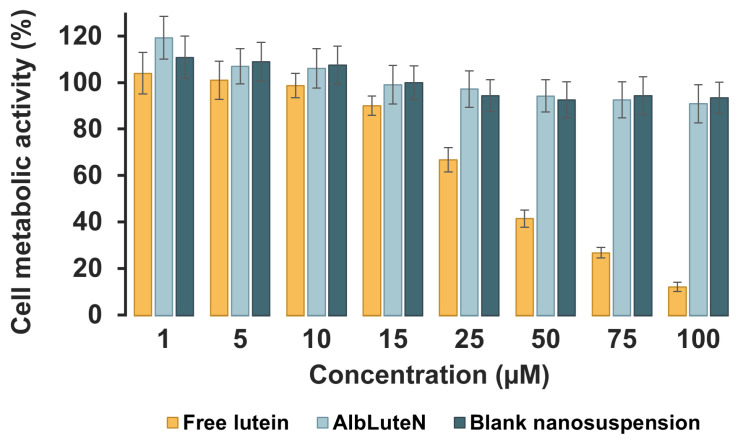
Effect of free lutein, AlbLuteN, and blank albumin nanosuspension on the metabolic activity of THLE-2 human hepatocytes after 48 h of exposure, assessed by the MTT assay. Free lutein and AlbLuteN were tested at equivalent lutein concentrations (1–100 µM), while the blank nanosuspension was applied at carrier concentrations matching those in AlbLuteN. Cell metabolic activity is expressed as a percentage of the untreated control. Data are presented as mean ± SEM (*n* = 3).

The effect of free lutein, AlbLuteN, and the blank albumin nanosuspension on cell metabolic activity was evaluated in THLE-2 human hepatocytes using the MTT assay following 48 h of exposure (Figure 9).

Free lutein exhibited a clear concentration-dependent cytotoxic effect. At concentrations up to 10 µM, cell metabolic activity remained close to control levels, whereas a decrease below 50% was observed at concentrations >25 µM. In contrast, AlbLuteN did not induce a pronounced reduction in cell metabolic activity over the entire concentration range tested. Cell metabolic activity remained above 90% at all concentrations up to 100 µM, indicating high cytocompatibility of the albumin-based nanoformulation. The blank albumin nanosuspension showed similar effects, confirming the carrier system’s intrinsic biocompatibility. Notably, encapsulation of lutein within the albumin matrix markedly attenuated the cytotoxic effects observed for the free compound at higher concentrations. This protective effect may be related to the controlled presentation of lutein to cells and to reduced local exposure to high free lutein concentrations.

The observed cytocompatibility of AlbLuteN is consistent with the biological role of albumin and supports the suitability of this nanocarrier for parenteral delivery of poorly water-soluble bioactives. Taken together with the hemocompatibility data, these results demonstrate that AlbLuteN exhibits a highly favorable biocompatibility profile in both blood and hepatic cells, supporting its further evaluation under conditions relevant to PN-associated liver injury.

Despite the encouraging results obtained in the present study, several limitations should be considered. The biological effects of lutein were evaluated exclusively in an in vitro hepatocyte model, which cannot fully replicate the complex multicellular and systemic processes underlying IFALD in vivo. Moreover, the applied experimental design reflects a simplified in vitro exposure model and does not capture the temporal progression and disease heterogeneity characteristic of IFALD, a spectrum of liver disorders evolving over time. In addition, the present study focused primarily on the modulation of selected signaling pathways and the formulation biocompatibility assessment, without addressing the cellular uptake, intracellular fate, or lutein release kinetics from the nanoformulation. Importantly, the in vivo pharmacokinetics, biodistribution, and organ-specific accumulation following intravenous administration were not evaluated. Future studies could therefore include evaluation of lutein in more complex experimental systems, such as co-culture or three-dimensional hepatic models, as well as in vivo models of PN-associated liver injury. Moreover, detailed analyses of cellular uptake, in vivo therapeutic efficacy, biodistribution, and pharmacokinetics may further help define the translational potential of AlbLuteN.

## 3. Materials and Methods

### 3.1. Materials

Lutein (pharmaceutical secondary standard) was obtained from Merck KGaA (Darmstadt, Germany). LPS was purchased from Sigma-Aldrich (Saint Louis, MO, USA), and Intralipid was obtained from Fresenius Kabi AB (Uppsala, Sweden). Human serum albumin (Albiomin) was supplied by Biotest Pharma GmbH (Dreieich, Germany). Water for injection and 0.9% sodium chloride solution were obtained from B. Braun Melsungen AG (Melsungen, Germany). Organic solvents of high-performance liquid chromatography (HPLC) grade were procured from Avantor Performance Materials Poland S.A. (Gliwice, Poland).

### 3.2. Cell Culture and Treatment

Human immortalized hepatocytes THLE-2 (ATCC CRL-2706) were obtained from the American Type Culture Collection (ATCC, Manassas, VA, USA). Cells were cultured in Bronchial Epithelial Growth Medium supplemented with the BulletKit (Lonza, Cologne, Germany), 10% fetal bovine serum, 5 ng/mL epidermal growth factor, and 70 ng/mL phosphoethanolamine at 37 °C in a humidified atmosphere containing 5% CO_2_.

To evaluate IFALD-related inflammatory and metabolic stress, we employed our previously described IFALD model in which THLE-2 cells were exposed to LPS (0.1 µg/mL), Intralipid (10 mg/mL), or both for 24 h [21]. For combined treatment, cells were first incubated with Intralipid for 1 h, followed by the addition of LPS for a further 24 h of incubation. To assess the effect of lutein, the compound was added at two selected concentrations simultaneously with LPS after the initial 1 h exposure to Intralipid. Control cells were maintained in complete culture medium without treatment.

### 3.3. MTT Assay

Cell metabolic activity as an indicator of cell viability was assessed using the MTT assay according to a standard protocol. Briefly, THLE-2 cells were seeded at a density of 1 × 10^4^ cells per well in 96-well plates and allowed to adhere for 24 h. Cells were then treated with (i) Intralipid at concentrations of 0.5–15 mg/mL, (ii) LPS at concentrations of 0.0125–1 µg/mL, or (iii) free lutein at concentrations of 1–100 µM and incubated for 24 h to identify non-cytotoxic concentrations for subsequent experiments. After treatment, cells were washed twice with phosphate-buffered saline and incubated for 4 h with culture medium containing 0.5 mg/mL MTT. The resulting formazan crystals were dissolved in acidic isopropanol, and absorbance was measured at 570 nm and 690 nm.

For evaluation of nanoformulation effects on cell metabolic activity, the MTT assay was performed as described above, except that cells were treated with free lutein, AlbLuteN, or blank albumin nanosuspension at concentrations corresponding to 1–100 µM lutein and incubated for 48 h. The lutein concentration was equivalent in samples containing free lutein and AlbLuteN, while the blank nanosuspension was applied at carrier concentrations matching those in AlbLuteN.

### 3.4. Total Protein Lysates Preparation

Lysates were prepared using Radioimmunoprecipitation assay buffer with the addition of protease inhibitors (Sigma-Aldrich, USA). Protein concentration was assessed, and the samples were stored at −80 °C for future downstream applications.

### 3.5. Bead-Based Multiplex Immunoassay (MAGPIX)

The levels of selected total and phosphorylated signaling proteins were quantified using a magnetic bead–based multiplex immunoassay on the Luminex MAGPIX system (Luminex Corporation, Austin, TX, USA) according to the manufacturer’s instructions. Cell lysates were prepared from THLE-2 cells and analyzed using a high-sensitivity magnetic bead panel (Merck, Darmstadt, Germany), enabling the simultaneous detection of p38, p-p38, JNK, p-JNK, ERK1/2, p-ERK1/2, NF-κB, p-NF-κB, Akt, p-Akt, STAT3, p-STAT3, STAT5, p-STAT5, p70S6K, p-p70S6K, CREB, and p-CREB.

Briefly, lysates were suspended in MILLIPLEX MAP assay buffer and incubated with the bead suspension in 96-well plates overnight at 2–8 °C on a shaker protected from light. After washing, detection antibodies were added and incubated for 1 h at room temperature, followed by the addition of streptavidin–phycoerythrin. Signal amplification was performed using the MILLIPLEX MAP amplification buffer according to the manufacturer’s protocol. Beads were then resuspended in assay buffer and analyzed using the Luminex MAGPIX® platform (Luminex Corporation, Austin, TX, USA).

Fluorescence signals were acquired and processed using xPonent 4.2 software (Luminex Corporation), and data were further analyzed with MILLIPLEX Analyst 5.1 software (EMD Millipore, Burlington, MA, USA). Protein levels were expressed as mean fluorescence intensity and normalized to untreated control cells.

### 3.6. RNA Extraction, cDNA Synthesis, and qPCR

Total RNA was isolated using the GeneMatrix Universal DNA/RNA/Protein Purification Kit (EURx, Gdańsk, Poland). Reverse transcription was subsequently performed with the RevertAid First Strand cDNA Synthesis Kit (Thermo Fisher Scientific, Waltham, MA, USA) according to the manufacturer’s protocol. qPCR was carried out using the Maxima SYBR Green Kit (Fermentas Inc., Waltham, MA, USA) on a LightCycler thermal cycler (Roche, Penzberg, Germany). The amplification program consisted of an initial enzyme activation step at 95 °C for 5 min, followed by 40 cycles of denaturation at 95 °C for 15 s, annealing at 56 °C for 20 s, and extension at 72 °C for 40 s, with a final elongation step at 72 °C for 5 min. Melting curve analysis was performed to confirm amplicon specificity. Gene expression levels were normalized to *TBP* (TATA box binding protein) and *PBGD* (porphobilinogen deaminase). Relative expression was calculated using the Pfaffl method. Primer sequences are provided in Table 1. Primers were designed using Beacon Designer software 7.9, verified by BLAST+ 2.17.0 analysis to minimize non-specific binding, and synthesized at the Institute of Biochemistry and Biophysics (Warsaw, Poland). Only primer pairs producing intron-spanning amplicons were used.

### 3.7. AlbLuteN Preparation

AlbLuteN was prepared using a modified nab™ technology as described previously [22] with minor modifications. Briefly, a human serum albumin solution (40 mg/mL) was prepared by diluting Albiomin (200 g/L) with water for injection to a final volume of 10 mL. Subsequently, 1 mL of lutein dissolved in dichloromethane (25 mg/mL) was added to the albumin solution. The mixture was placed in an ice bath and subjected to probe sonication using a titanium alloy probe (type KE76, diameter 6 mm) (Sonopuls HD 2070 ultrasonic homogenizer, Bandelin Electronic GmbH & Co. KG, Berlin, Germany) for 3 min at 80% amplitude in pulsed mode (15 s on/15 s off) to form an oil-in-water emulsion. The resulting emulsion was transferred to a round-bottom flask, and the organic solvent was removed under reduced pressure using a rotary evaporator at 40 °C for 30 min. The obtained nanoparticle suspension was subsequently freeze-dried without cryoprotectant under conditions described in the literature [69] to yield a solid formulation. A blank nanosuspension was prepared following the same procedure, without the addition of lutein.

### 3.8. AlbLuteN Physicochemical Characterization

The Z-average and PDI of AlbLuteN were determined by dynamic light scattering using a Zetasizer Nano ZS (Malvern Instruments, Malvern, UK). Measurements were performed at 25 °C after reconstitution of the freeze-dried formulation and 100-fold dilution with water for injection. The zeta potential was measured by laser Doppler electrophoresis with the same instrument and calculated using the Smoluchowski equation. The morphology of the nanoparticles was studied by Mira 3 FEG SEM (Tescan, Brno, Czech Republic) equipped with an In-Beam SE detector. For SEM analysis, drops of the nanoparticle suspension were placed onto aluminum microscope stages, dried in a desiccator at room temperature, and carbon-coated prior to imaging.

The lutein content in AlbLuteN was determined by HPLC with diode-array detection (HPLC–DAD) using a previously described method [22]. Freeze-dried samples were reconstituted (10 mg in 1 mL of water) and mixed with an organic solvent mixture of methanol and acetonitrile (1:1, *v*/*v*) at a volume ratio of 1:25 (hydrated sample:solvent mixture). The samples were bath-sonicated for 30 min, cooled for an additional 30 min at 4 °C, and centrifuged at 12,000 rpm for 15 min at 4 °C. The supernatants were filtered through 0.2 µm syringe filters prior to analysis.

HPLC–DAD analysis was performed using an Infinity II 1260 system (Agilent Technologies, Santa Clara, CA, USA) equipped with a LiChrospher^®^ 100 RP-18 endcapped column (250 mm × 4 mm, 5 µm). The mobile phase consisted of methanol–acetonitrile (90:10, *v*/*v*) supplemented with triethylamine (9 µM) [70]. The flow rate was 1.5 mL/min, the column temperature was set to 30 °C, the injection volume was 20 µL, and detection was carried out at 475 nm. Drug loading was calculated as the percentage of lutein content relative to the total mass of the lyophilizate.

### 3.9. Hemolysis Assay

Red blood cell concentrate was obtained from a blood donation center; ethical approval was not required for this study.

The hemocompatibility of AlbLuteN was evaluated using a hemolysis assay based on the protocol proposed by Sæbø et al. [71], with minor modifications. A 1% erythrocyte suspension was prepared from a red blood cell concentrate and mixed with the tested samples at a volume ratio of 1:1. The tested conditions included a positive control (10% Triton X-100), a negative control (0.9% NaCl), and AlbLuteN reconstituted in 0.9% NaCl to concentrations of 10 and 50 µg/mL, corresponding to the total mass of the nanoformulation rather than the lutein content. In parallel, erythrocyte-free samples containing the corresponding AlbLuteN concentrations were prepared to correct for background absorbance originating from the nanoparticle formulation. All samples were incubated for 1 h at 37 °C under gentle agitation. After incubation, samples were centrifuged at 4500 rpm for 5 min, and the absorbance of the supernatants was measured at 577 nm using an Agilent Cary 3500 Compact UV–Vis spectrophotometer (Agilent Technologies, Santa Clara, CA, USA). This wavelength was selected to minimize background absorbance originating from the nanoparticles.

% hemolysis was calculated based on Equation (1):

(1) $$\%\;hemolysis=\frac{A(sample)-A(background)-A(negative)}{A(positive)-A(negative)},$$

where *A*(sample) is the absorbance of AlbLuteN samples incubated with erythrocytes, *A*(background) is the absorbance of erythrocyte-free samples containing the corresponding AlbLuteN concentrations, *A*(negative) is the absorbance of the negative control (0.9% NaCl), and *A*(positive) is the absorbance of the positive control (10% Triton X-100).

To assess potential assay interference related to hemoglobin–nanoparticle interactions, a hemoglobin binding/co-sedimentation control was prepared by mixing 1490 µL of the positive control after 1 h incubation with 10 µL of either 0.9% NaCl or AlbLuteN (at a final concentration corresponding to the 50 µg/mL sample). The solutions were well mixed, centrifuged under the same conditions as the tested samples, and the absorbance of the supernatants at 577 nm was measured.

### 3.10. Statistical Analysis

Statistical analysis was performed using GraphPad Prism version 11.0.0 (GraphPad Software, San Diego, CA, USA). All experiments were performed at least in triplicate, and data are presented as mean ± SEM for MTT, MAGPIX, and qPCR results, or as mean ± SD for AlbLuteN preparation, physicochemical characterization, and hemolysis data. For comparisons between two groups, Student’s *t*-test was used. For multiple group comparisons, one-way analysis of variance (ANOVA) followed by Dunnett’s post hoc test was applied. A *p*-value < 0.05 was considered statistically significant.

## 4. Conclusions

A human hepatocyte–based in vitro model of IFALD was established using THLE-2 cells exposed to defined inflammatory (LPS) and lipid-derived (soybean oil–based lipid emulsion) stressors associated with intestinal failure and PN. This model provided mechanistic insight into the combined impact of endotoxin and omega-6–rich lipid emulsions on hepatocellular inflammatory and metabolic signaling, complementing existing data obtained from non-human systems and transformed hepatoma cell lines. The model was subsequently used to assess the effects of lutein on hepatocellular signaling under IFALD-like conditions. Lutein modulated dysregulated signaling pathways associated with inflammatory activation and metabolic stress, supporting its anti-inflammatory and metabolic regulatory potential. To address practical considerations for lutein delivery in patients on PN, a human serum albumin–based lutein nanoformulation intended for intravenous administration was evaluated for biocompatibility. The nanoformulation exhibited hemocompatibility and cytocompatibility, possibly stabilizing erythrocytes and showing no cytotoxic effects in human THLE-2 hepatocytes, thereby supporting its suitability for systemic administration. Together, these findings provide a biological and technological rationale for further investigation of lutein-based adjunctive strategies to mitigate IFALD in patients receiving PN.

## Figures and Tables

**Table 1:** Primer sequences used for qPCR reactions.

Primer		Sequence	Product Size (bp)
*PBGD*	forward	5′CCGCATCTGGAGTTCAGGAGTATTC	101
	reverse	5′CCAGCTGTTGCCAGGATGATG	
*TBP*	forward	5′GGCACCACTCCACTGTATC	183
	reverse	5′GGGATTATATTCGGCGTTTCG	
*ABCA1*	forward	5′TGAGGGAACATGGCTTGTT	143
	reverse	5′CTCAGCCGAACAGAGATCAG	
*SREBF2*	forward	5′AACGGTCATTCACCCAGGTC	133
	reverse	5′GGCTGAAGAATAGGAGTTGCC	
*CYP7A1*	forward	5′CATTTGGGCACAGAAGCATTG	174
	reverse	5′AGGCAGCGGTCTTTGAGTTAG	
*PRKAA2*	forward	5′TCAATCGTTCTGTCGCCAC	530
	reverse	5′ATACGGTTTGCTCTGACTTCG	
*HMGCR*	forward	5′TTCTTGCCAACTACTTCGTGTT	102
	reverse	5′GCTGCCAAATTGGACGACC	

## Data Availability

The raw data supporting the conclusions of this article will be made available by the authors on request.

## Abbreviations

The following abbreviations are used in this manuscript:

AA	Arachidonic acid
ABCA1	ATP-binding cassette subfamily A member 1
Akt	Protein kinase B
AlbLuteN	Albumin–lutein nanosuspension
AMPKα2	AMP-activated protein kinase α2
COX-2	Cyclooxygenase-2
CREB	cAMP response element-binding protein
CYP7A1	Cholesterol 7 α-hydroxylase
ERK1/2	Extracellular signal–regulated kinases 1 and 2
FAs	Fatty acids
FXR	Farnesoid X receptor
HDL	High-density lipoprotein
HMGCR	3-hydroxy-3-methylglutaryl-CoA reductase
HPLC-DAD	High-performance liquid chromatography with diode-array detection
IFALD	Intestinal failure–associated liver disease
IL-1β	Interleukin-1β
IL-6	Interleukin-6
IRS1	Insulin receptor substrate 1
JNK	c-Jun N-terminal kinase
LPS	Lipopolysaccharide
MAPKs	Mitogen-activated protein kinases
MTT	3-(4,5-dimethylthiazol-2-yl)-2,5-diphenyltetrazolium bromide
NF-κB	Nuclear factor κB
Nrf2	Nuclear factor erythroid 2-related factor 2
PBGD	Porphobilinogen deaminase
PDI	Polydispersity index
PI3K	Phosphoinositide 3-kinase
PN	Parenteral nutrition
p70S6K	70 kDa ribosomal protein S6 kinase
qPCR	Quantitative real-time polymerase chain reaction
SEM	Scanning electron microscopy
SREBP2	Sterol regulatory element-binding protein 2
STAT3	Signal transducer and activator of transcription 3
STAT5	Signal transducer and activator of transcription 5
TBP	TATA box binding protein
TLR4	Toll-like receptor 4
Z-average	Intensity-weighted mean hydrodynamic diameter
